# Stroke Knowledge and Response Among the General Population in Saudi Arabia: A Population-Based Survey

**DOI:** 10.7759/cureus.65587

**Published:** 2024-07-28

**Authors:** Wejdan M Bajandouh, Turki N Alotaibi, Abdullah R Alharbi, Saad A Alzahrani, Ghidaa A Alghamdi, Ahmad H Aleissi, Asem Rashed, Mokhtar Shatla

**Affiliations:** 1 Medicine and Surgery, College of Medicine, Umm Al-Qura University, Makkah, SAU; 2 Neurology, King Abdullah Medical City, Makkah, SAU; 3 Emergency Medicine, King Abdullah Medical City, Makkah, SAU; 4 Medicine, College of Medicine, Umm Al-Qura University, Makkah, SAU

**Keywords:** recognition of stroke symptoms, saudi arabia, health education, public response, stroke awareness

## Abstract

Introduction: Stroke, a condition that disrupts brain function and can result in long-lasting clinical symptoms or death, remains a substantial global health concern. General awareness of stroke leads to a proper response to stroke, pursuit of medical treatment, and a better outcome. Our study aims to explore how the general Saudi Arabian population responds to stroke, investigate the relationship between stroke knowledge and appropriate actions, and identify the most relied upon medical information source about stroke among the public. By highlighting these aspects, we aspire to contribute to the development of effective stroke prevention and management strategies in Saudi Arabia. Methodology: This descriptive, cross-sectional, web-based study was carried out across multiple regions in Saudi Arabia. Data collection involved a validated online questionnaire (STAT) in Arabic, targeting adults aged 18 and older. Data analysis was performed using SPSS software.

Results: The study included 390 participants. Notable findings emerged regarding the recognition of stroke symptoms and the factors influencing this recognition. A substantial proportion of respondents correctly identified visual disturbances (55.9%), motor impairment (39.5%), cardiac symptoms (56.2%), sensory symptoms (32.8%), chest pain (62.6%), and arm weakness (36.7%) as indicators requiring immediate medical attention. However, there were lower correct response rates for symptoms such as sudden dizziness (22.8%), confusion (16.9%), difficulty understanding (27.9%), and urinary symptoms (26.9%). Older individuals and retirees generally scored higher in correctly identifying these symptoms.

Conclusion: Our study highlights a commendable level of awareness of certain stroke symptoms, such as visual disturbances and chest pain, among the general population of Saudi Arabia. Nonetheless, notable gaps remain in recognizing other symptoms, such as sudden dizziness and confusion. This identification gap presents a clear opportunity for targeted educational initiatives that are essential to improving public recognition of these symptoms. By addressing these gaps, we aim to enhance the response to stroke incidents, potentially improving outcomes and reducing morbidity rates.

## Introduction

A stroke is an episode of acute neurological dysfunction presumed to be caused by ischemia or hemorrhage, persisting ≥24 hours or until death [1]. Stroke is the world’s second leading cause of death following myocardial infarction, with an annual mortality rate of 5.5 million [2]. In the United States, stroke ranks among the top causes of long-term disability, affecting 795,000 patients annually, 26% of whom become disabled and 50% of whom experience restricted mobility [3]. In Saudi Arabia, the prevalence of stroke was estimated at 40 per 100,000 annually in the Eastern province and 43.8 per 100,000 annually in Riyadh [4].

Community attitudes and knowledge of stroke symptoms are crucial for effective stroke prevention [5]. A lack of awareness about the warning signs of stroke and its critical indicators often leads to delays in seeking medical advice and indecision regarding hospital admission [5]. Greater awareness about the importance of timely medical intervention, from the onset of symptoms to hospital arrival, can substantially improve treatment outcomes and reduce comorbidities. Additionally, appropriate lifestyle changes can substantially lower the risk of stroke-related morbidity and mortality [5-8].

The primary factor contributing to adult neurological impairment is the delay in hospital presentation, which poses the most substantial prehospital barrier to thrombolysis within 4.5 hours of the onset of stroke symptoms [9,10]. Education about stroke and the urgency of seeking immediate medical help can enhance public awareness and potentially improve outcomes [11,12].

Previous studies in Saudi Arabia, Lebanon, Iran, Spain, and China have identified heart disease, hypertension, dyslipidemia, smoking, old age, and obesity as the most recognized risk factors for stroke [13]. Slurred speech and sudden weakness in the face, arms, or legs were identified as the most recognizable warning signs [13]. Most people would call emergency services if they suspected a stroke [13,14]. Factors such as urban residency, high economic status, younger age, personal acquaintance with stroke victims, and a higher level of education have been associated with better knowledge about stroke and more effective responses [13,15]. The ability to recognize multiple stroke symptoms has been directly linked to quicker emergency medical responses post-stroke [13,14,16]. Conversely, delayed contact with emergency services and hospital arrivals have been associated with a failure to recognize stroke symptoms, low educational levels, unemployment, rural residency, younger age (40-59 years), and low income [17,18].

## Materials and methods

Study design

This web-based descriptive cross-sectional study was conducted across several regions in Saudi Arabia.

Sample size

The minimum sample size for this study is 385 and was calculated using OpenEpi version 3.0, a widely used and validated method in epidemiological research [19]. Given that Saudi Arabia’s estimated total population in 2022 is approximately 36,184,858, according to the Worldometer website, we maintained a confidence interval (CI) level of 95%, assumed an expected frequency percentage of 50%, and set the design effect at one. The final sample size is 390 participants.

Sampling techniques

The study utilized a questionnaire designed to be brief, straightforward, and easily comprehensible by the general public (Tables 5, 6 of Appendices). The items were selected based on a comprehensive literature review. An online survey was conducted in Arabic using Google Forms, employing the snowball sampling method. Electronic links detailing the survey’s objectives and target audience, along with taking protections to ensure confidentially, as well as an invitation for voluntary participation, were sent to potential respondents. The survey was disseminated electronically to eligible populations via social media applications: send the link via WhatsApp, Instagram, or X, following clearance from the Umm Al-Qura University institutional review board.

Data collection methods, instruments used, and measurements

Data were collected using a validated online questionnaire (STAT) in Arabic. The questionnaire comprised several sections: 1) consent form; 2) sociodemographic data; 3) assessment of knowledge about stroke; 4) assessment of response to stroke; and 5) to ensure confidentiality, a system combining codes, numbers, and pseudonyms was established. Only the researchers had access to the data.

Data management and analysis plan

The data were analyzed using SPSS version 27.0.1. Descriptive statistics summarized the data, displaying numerical data as mean ± SD or depending on the distribution, median and range. Percentages and frequency values described categorical variables. Group comparisons were conducted using the Mann-Whitney test and the student t-test. The chi-squared test assessed associations between categorical variables. The significance threshold was set at a p-value of less than 0.05, with a CI of 95%.

Statistical analysis

The data were analyzed using IBM SPSS version 27.0.1. Continuous data were assessed using descriptive statistics, including means, SDs, medians, and IQR. Frequency distributions were utilized to summarize categorical data, encompassing socioeconomic and demographic characteristics.

The scoring system, informed by expert consensus and validated stroke knowledge evaluation tools, quantified the participants’ understanding of appropriate responses to stroke symptoms. Each correct answer was assigned one point, with possible scores ranging from 0 to 28. This system helped standardize and simplify the evaluation of stroke symptom knowledge among the participants.

Before analysis, the normality of continuous data, such as scores for correct answers, was evaluated using the non-parametric Kolmogorov-Smirnov test. This test determines whether a sample’s distribution matches a specific population distribution. Due to the non-normal distribution of the variables, non-parametric tests were applied to analyze differences in mean scores across socioeconomic and demographic attributes. The Kruskal-Wallis test was employed for variables with more than two categories, such as age, education level, employment status, city of residence, and living location. Meanwhile, the Mann-Whitney U test was used for dichotomous variables, such as gender.

The significance thresholds for all statistical tests were set at p < 0.05. A result was considered statistically significant if the p-value was less than 0.05, indicating a low probability that the observed differences were due to chance. This threshold is commonly used in scientific studies to determine the presence of a substantial effect.

## Results

The sociodemographic characteristics of the participants in this study display a diverse profile. The age distribution shows that the majority, 61.0%, fall within the 18-44 age bracket, while those aged 45-64 comprise 23.3%. A smaller segment includes individuals under 18 (12.8%) and those over 65 (2.8%). Gender distribution indicates a predominance of females, who constitute 77.4% of the participants, with males making up the remaining 22.6%. In terms of education, the highest proportion of participants hold a Bachelor’s degree (60.0%), followed by those with a high school education or less (33.3%) and postgraduates (6.7%). Employment status is varied, with students representing a substantial portion (36.7%), alongside unemployed individuals (29.2%), employees (26.4%), and retirees (7.7%). Demographic questions have been used to categorize according to the region, most participants reside in the Western Region (54.9%), followed by the Central (20.8%) and Eastern (12.8%) regions, with smaller percentages in the Southern (9.2%) and Northern (2.3%) regions. Demographic questions have been used to classify the place, the vast majority live in urban areas (90.5%), while smaller proportions are in rural areas (6.2%) or suburbs (3.3%). Overall, these findings offer insights into various demographic segments within the population and reflect a representative sample (Table 1).

**Table 1:** Sociodemographic Characteristics of Participants

		N	%
Age	18-44	238	61.0%
	45-64	91	23.3%
	Under 18	50	12.8%
	Above 65	11	2.8%
Gender	Female	302	77.4%
	Male	88	22.6%
Education level	Bachelor’s	234	60.0%
	High school or less	130	33.3%
	Postgraduate	26	6.7%
Employment status	Student	143	36.7%
	Unemployed	114	29.2%
	Employee	103	26.4%
	Retired	30	7.7%
City of residence	Western region	214	54.9%
	Central region	81	20.8%
	Eastern region	50	12.8%
	Southern region	36	9.2%
	Northern region	9	2.3%
Place of residence	City	353	90.5%
	Rural area	24	6.2%
	Suburbs	13	3.3%

Response toward stroke symptoms

The data indicate that 55.9% of participants promptly recognized the necessity of immediate medical attention when experiencing sudden difficulty seeing in one eye. This underscores a commendable awareness of visual disturbances as potential indicators of stroke. Additionally, 39.5% of respondents acknowledged the urgency when someone became unable to perform simple tasks, such as retrieving keys from a purse, highlighting motor impairments often associated with neurological conditions like stroke.

Furthermore, 56.2% of the participants identified the need for immediate medical intervention when experiencing sensations of heart fluttering and skipping beats. Notably, 32.8% recognized the significance of tingling sensations in the left leg, indicative of potential neurological involvement and a precursor to more severe stroke manifestations.

Moreover, 62.6% of participants accurately perceived the severity of chest pain, described as feeling like an elephant sitting on the chest, a classic symptom of heart attacks. In addition, 36.7% correctly identified sudden weakness in one arm as a critical sign of stroke, reflecting an awareness of the motor deficits characteristic of this condition.

In the first section of the table, sudden dizziness prompted immediate action in only 22.8% of cases, suggesting a potential lack of recognition of vertigo as a symptom of stroke (Table 2).

**Table 2:** First Section of Participants’ Responses to Stroke Symptoms *Correct response

Response description	Call 991 or go to the emergency room immediately*		Call your doctor’s office immediately		I wait an hour, then decide		I wait one day, then decide	
	N	%	N	%	N	%	N	%
Sudden difficulty seeing in one eye	218	55.9%	45	11.5%	90	23.1%	37	9.5%
She suddenly became clumsy and was unable to retrieve her keys from her purse	154	39.5%	81	20.8%	90	23.1%	65	16.7%
Unexpected irregular heartbeat	213	54.6%	69	17.7%	82	21.0%	26	6.7%
She experienced double vision, vertigo, and nausea while watching TV and required support	199	51.0%	94	24.1%	73	18.7%	24	6.2%
Sudden numbness in the leg, predominantly on one side	141	36.2%	71	18.2%	130	33.3%	48	12.3%
Sudden onset of dizziness	89	22.8%	68	17.4%	154	39.5%	79	20.3%
Temporary language comprehension issues; it seemed as if others were speaking a foreign language	133	34.1%	67	17.2%	123	31.5%	67	17.2%
Numbness in the right hand during weightlifting, though he completed the exercise	93	23.8%	84	21.5%	121	31.0%	92	23.6%
Pain in the knuckles and a spasmodic finger that could not be extended	169	43.3%	91	23.3%	92	23.6%	38	9.7%
Impaired speech as if intoxicated, despite no alcohol consumption	217	55.6%	72	18.5%	76	19.5%	25	6.4%
She appeared pale and reported sensations of a fluttering and skipping heartbeat	219	56.2%	82	21.0%	69	17.7%	20	5.1%
Tingling in the left leg while watching TV, as if the leg was falling asleep	128	32.8%	75	19.2%	150	38.5%	37	9.5%
Intense chest pressure described as feeling like an “elephant sitting on my chest”	244	62.6%	72	18.5%	59	15.1%	15	3.8%
Sudden weakness in the arm, particularly on one side	143	36.7%	90	23.1%	114	29.2%	43	11.0%

Transitioning to the second section of the table, symptoms eliciting relatively lower correct responses include sudden confusion and difficulty understanding. Sudden confusion prompted immediate action in only 16.9% of cases, indicating a possible underestimation of cognitive impairments as stroke symptoms. Similarly, difficulty understanding was recognized by only 27.9% of respondents, suggesting a lesser awareness of cognitive deficits in stroke presentations. Furthermore, sudden problems with speaking prompted correct responses from 42.3% of participants.

A frequent urge to urinate, accompanied by a burning sensation and cloudy urine, which are urinary symptoms that may complicate stroke, prompted immediate action in only 26.9% of cases, suggesting a relatively reasonable understanding of the urinary symptoms that are not an initial symptom to watch out for in stroke occurrence.

Finally, sudden facial weakness, particularly on one side, led 52.1% of participants to seek immediate medical attention, demonstrating an awareness of facial asymmetry as a critical sign of stroke (Table 3).

**Table 3:** Second Section of the Participants’ Responses to Stroke Symptoms *Correct response

	Call 991 or go to the emergency room immediately*		Call your doctor’s office immediately		I wait an hour, then decide		I wait one day, then decide	
	N	%	N	%	N	%	N	%
He struggled to eat lunch as pieces of his sandwich fell from the right side of his mouth	207	53.1%	78	20.0%	64	16.4%	41	10.5%
Sudden confusion	66	16.9%	57	14.6%	131	33.6%	136	34.9%
Dizziness accompanied by sudden vision problems	108	27.7%	87	22.3%	135	34.6%	60	15.4%
Sudden difficulty understanding	109	27.9%	84	21.5%	122	31.3%	75	19.2%
Frequent urge to urinate, burning sensation during urination, and cloudy urine	105	26.9%	119	30.5%	58	14.9%	108	27.7%
Inability to reach for my bag due to paralysis of the right arm, accompanied by drooling and garbled speech	282	72.3%	59	15.1%	36	9.2%	13	3.3%
Sudden, severe headache without a known cause	116	29.7%	92	23.6%	109	27.9%	73	18.7%
Sudden difficulty speaking	165	42.3%	80	20.5%	117	30.0%	28	7.2%
Chest pain persisting for more than a few minutes or worsening	222	56.9%	72	18.5%	71	18.2%	25	6.4%
He repeatedly covered and uncovered his eyes, blinking, and complained of temporary vision loss	122	31.3%	96	24.6%	91	23.3%	81	20.8%
Sudden weakness of the face, predominantly on one side	203	52.1%	91	23.3%	65	16.7%	31	7.9%
Sudden weakness in the arm and face, particularly on one side, with speech difficulties	246	63.1%	80	20.5%	45	11.5%	19	4.9%
Sudden immobility of the right arm	223	57.2%	84	21.5%	67	17.2%	16	4.1%
Sudden loss of coordination	166	42.6%	76	19.5%	98	25.1%	50	12.8%

Factors associated with correct response knowledge

The study examined participants’ responses to stroke symptoms, assessed through a correct response score ranging from 0 to 28. The analysis revealed variations in mean scores across demographic and socioeconomic factors. Participants aged over 65 had the highest mean score (15.73, SD = 8.36), followed by retirees (15.47, SD = 7.11), suggesting a possibly greater awareness among these older age groups. However, the differences in mean scores among age groups were not statistically significant (p = 0.275).

Employed individuals recorded a substantially higher mean score (13.41, SD = 8.22) compared to unemployed participants (11.78, SD = 6.96), with a statistically significant difference (p = 0.002). Postgraduate students achieved the highest mean score among educational levels (14.46, SD = 7.70). No substantial differences were noted concerning education level (p = 0.262), gender (p = 0.995), city of residence (p = 0.439), and rural versus urban living (p = 0.643) (Table 4).

**Table 4:** Factors Associated With Knowledge of Correct Responses ^K/U^Kruskal-Wallis test for independent samples; Mann–Whitney U Test *p < 0.05, significant

		Correct response score (0-28)				
		Mean	SD	Median	IQR	P-value^K/U^
Age	18-44	11.61	7.30	11.00	6.00-16.00	0.275
	45-64	12.66	7.62	11.00	7.00-17.00	
	Above 65	15.73	8.36	16.00	11.00-21.00	
	Under 18	12.24	8.60	11.00	6.00-16.00	
Gender	Female	12.05	7.65	11.00	6.00-16.00	0.995
	Male	12.07	7.41	11.00	7.00-16.00	
Education level	Bachelor’s	11.75	7.40	11.00	6.00-16.00	0.262
	High school or less	12.12	7.86	11.00	6.00-15.00	
	Postgraduate	14.46	7.70	15.50	8.00-19.00	
Employment status	Employee	13.41	8.22	12.00	8.00-19.00	0.002*
	Retired	15.47	7.11	15.00	9.00-22.00	
	Student	10.57	7.36	9.00	6.00-14.00	
	Unemployed	11.78	6.96	11.00	6.00-16.00	
City of residence	Central region	12.36	7.39	11.00	7.00-16.00	0.439
	Eastern region	10.50	7.49	9.00	6.00-13.00	
	Southern region	12.19	9.88	10.50	4.00-19.50	
	Northern region	12.44	5.73	14.00	7.00-17.00	
	Western region	12.26	7.34	11.00	7.00-16.00	
Place of residence	City	12.11	7.50	11.00	7.00-16.00	0.643
	Rural area	11.92	9.32	10.00	4.50-21.50	
	Suburbs	10.62	6.86	9.00	7.00-12.00	

## Discussion

This study has provided valuable insights into public awareness and response to stroke symptoms in Saudi Arabia. Although there is a general understanding of some stroke indicators, a substantial disparity remains in recognizing less common symptoms, potentially delaying crucial medical interventions.

Encouragingly, 55.9% of participants were able to correctly identify sudden difficulty seeing in one eye as a reason to seek medical attention. This high recognition rate suggests a robust public awareness of visual disturbances as a medical emergency, which is vital for timely medical responses [10]. Additionally, the fact that 39.5% of respondents acknowledged clumsiness, such as difficulty retrieving keys from a purse, underscores the recognition of motor impairments often linked with neurological conditions like stroke [20].

The data also show that 56.2% of participants understood heart fluttering as a sign necessitating immediate medical attention, demonstrating an understanding of cardiac symptoms linked to stroke [21]. Moreover, the recognition of tingling sensations in the left leg by 32.8% of participants indicates an understanding of sensory symptoms that may signal neurological involvement, such as stroke [22].

Furthermore, 62.6% of the participants recognized severe chest pain as a symptom requiring urgent care. This recognition emphasizes and highlights the public’s knowledge and understanding of cardiac-related symptoms linked to strokes [23]. The identification of sudden weakness in one arm by 36.7% of respondents highlights awareness of motor deficits, which are characteristic signs of stroke [24].

The lower response rate of 22.8% for sudden dizziness points to a notable gap in recognizing vertigo as a stroke symptom, which could result in delayed medical attention [25]. Similarly, the fact that only 16.9% of participants would react immediately to sudden confusion and 27.9% to sudden difficulty understanding indicates a possible underestimation of them as stroke symptoms and a critical need for enhanced public education on cognitive impairments as stroke symptoms [26].

Highlighting awareness of speech disturbances as critical indicators of stroke by 42.3% of participants demonstrates some recognition of this symptom, yet it also suggests that there is considerable room for improving public knowledge [22].

Additionally, the relatively low recognition (26.9%) of urinary symptoms that require immediate medical attention highlights a substantial area where public awareness of the urinary symptoms is not an initial symptom to watch out for in stroke occurrence [27].

Previous studies in Saudi Arabia, Lebanon, Iran, Spain, and China have identified heart disease, hypertension, dyslipidemia, smoking, old age, and obesity as the most recognized risk factors for stroke [13]. Slurred speech and sudden weakness in the face, arms, or legs were identified as the most recognizable warning signs [13]. Most people would call emergency services if they suspected a stroke [13,14]. Factors such as urban residency, high economic status, younger age, personal acquaintance with stroke victims, and a higher level of education have been associated with better knowledge about stroke and more effective responses [13,15]. The ability to recognize multiple stroke symptoms has been directly linked to quicker emergency medical responses post-stroke [13,14,16]. Conversely, delayed contact with emergency services and hospital arrivals have been associated with a failure to recognize stroke symptoms, low educational levels, unemployment, rural residency, younger age (40-59 years), and low income [17,18].

Clinical implications and future research

Our study highlights the critical role of healthcare professionals in expanding public knowledge about stroke symptoms beyond the most commonly recognized signs [28]. By emphasizing lesser-known symptoms such as sudden dizziness, confusion, and urinary issues in educational campaigns, individuals can be better equipped to seek prompt medical intervention, potentially minimizing delays in the diagnosis and treatment of strokes [29]. Addressing these awareness gaps is essential for healthcare providers aiming to enhance stroke care outcomes and reduce the incidence of stroke-related disability and mortality.

Future research should focus on evaluating the impact of targeted educational interventions on improving the recognition and response to stroke symptoms across various demographic groups. Longitudinal studies are needed to measure the long-term effects of increased public awareness on stroke outcomes, including morbidity and mortality rates. Additionally, exploring the effectiveness of innovative approaches, such as digital health tools and community-based programs, in promoting stroke awareness and enabling timely medical response could offer crucial insights into optimizing stroke care delivery on a wider scale.

By advancing research in these areas, we can better develop evidence-based strategies to improve stroke prevention, treatment, and rehabilitation, thereby enhancing the overall quality of care in clinical settings.

Limitations

There are several limitations that must be acknowledged. The study relied on self-reported data, which might be susceptible to memory bias or participants’ misunderstanding of symptoms. Additionally, restricting the sample to a specific region or demographic could limit the applicability of the findings to larger populations. The cross-sectional methodology employed prevents establishing causal relationships between response knowledge and demographic characteristics. Despite these limitations, the study provides insightful information on the recognition of stroke symptoms and identifies areas requiring focused education and further research to improve stroke care outcomes.

## Conclusions

A substantial portion of the respondents correctly identified several key symptoms that require immediate medical attention, such as visual disturbances, motor impairment, and chest pain. However, there were noticeable gaps in awareness when it comes to symptoms like sudden dizziness, confusion, and urinary issues. Age and employment status were found to influence knowledge of stroke symptoms, with older individuals and those who were employed displaying higher levels of awareness. This highlights the need for targeted educational efforts aimed at improving public awareness and the recognition of stroke symptoms across different demographic groups. By doing so, we can ensure that timely intervention and better stroke care outcomes are achieved. Further research and community initiatives are necessary to address these knowledge gaps and promote proactive responses to stroke symptoms among the general population.

## Appendices

**Table 5:** STAT Questionnaire - English Version *Correct response

Call 991 or go to the emergency room immediately*	Call your doctor’s office immediately	I wait an hour, then decide	I wait one day, then decide	Response description
				Sudden difficulty seeing in one eye
				She suddenly became clumsy and was unable to retrieve her keys from her purse
				Unexpected irregular heartbeat
				She experienced double vision, vertigo, and nausea while watching TV and required support
				Sudden numbness in the leg, predominantly on one side
				Sudden onset of dizziness
				Temporary language comprehension issues; it seemed as if others were speaking a foreign language
				Numbness in the right hand during weightlifting, though he completed the exercise
				Pain in the knuckles and a spasmodic finger that could not be extended
				Impaired speech as if intoxicated, despite no alcohol consumption
				She appeared pale and reported sensations of a fluttering and skipping heartbeat
				Tingling in the left leg while watching TV, as if the leg was falling asleep
				Intense chest pressure described as feeling like an “elephant sitting on my chest”
				Sudden weakness in the arm, particularly on one side
				He struggled to eat lunch as pieces of his sandwich fell from the right side of his mouth
				Sudden confusion
				Dizziness accompanied by sudden vision problems
				Sudden difficulty understanding
				Frequent urge to urinate, burning sensation during urination, and cloudy urine
				Inability to reach for my bag due to paralysis of the right arm, accompanied by drooling and garbled speech
				Sudden, severe headache without a known cause
				Sudden difficulty speaking
				Chest pain persisting for more than a few minutes or worsening
				He repeatedly covered and uncovered his eyes, blinking, and complained of temporary vision loss
				Sudden weakness of the face, predominantly on one side
				Sudden weakness in the arm and face, particularly on one side, with speech difficulties
				Sudden immobility of the right arm
				Sudden loss of coordination

**Table 6:** STAT Questionnaire - Arabic Version *الاجابة الصحيحة

وصف الاستجابة	انتظر يوم واحد ثم اقرر	انتظر ساعة واحدة ثم اقرر	اتصل بمكتب الطبيب فورا	اتصل بالطوارئ ٩٩١*
صعوبة مفاجئة في الرؤية في عين واحدة				
أصبحت فجأة خرقاء. لم تستطع حتى الحصول على مفاتيحها من حقيبتها.				
نبضات قلب غير منتظمة مفاجأة .				
بينما كنا نشاهد التلفاز، قالت إنها بدأت ترى رؤية مزدوجة . أخبرتني أن الغرفة كانت تدور وشعرت بالغثيان. أمسكت ذراعي بشدة. إنها بالتأكيد لا تبدو في حالة سكر.				
خدر مفاجئ في الساق، وخاصة على جانب واحد.				
الدوخة المفاجئة.				
بدا الأمر وكأن الناس يتحدثون لغة أجنبية لبضع دقائق. لم أستطع فهمها ولم يبدو أنهم يفهمونني أيضا.				
اشتكى شريكي في صالة الألعاب الرياضية من إصابة يده اليمنى بالشعور بالخدر أثناء رفع الأثقال. كان قادرا على انهاء التمرين.				
كانت مفاصل أصابعه تؤلمه، خاصة بعد استخدام يديه. ثم تشنج إصبع من أصابعه فلم يستطع فتح يده.				
أجبت على الهاتف وأدركت أنني في حالة سكر. لم أستطع التحدث بوضوح بغض النظر عن مدى صعوبة محا ولتي. لم أشرب أي كحول.				
بدت شاحبة. وقالت إنها شعرت وكأن قلبها يرفرف ويتخطى النبض.				
بدأت ساقي اليسرى تنمل وأنا أشاهد التلفاز كان الأمر غريباً، كأن ساقي تغفو. حاولت فرك وهز ساقي دون جدوى.				
شعرت وكأن فيل كان جالسا على صدري. كان الألم يزيد.				
ضعف مفاجئ في الذراع خاصة على جانب واحد.				
رأيت أنه كان يحاول تناول طعام الغداء، لكن قطعة من الشطيرة ظلت تسقط من الجانب الأيمن من فمه.				
الارتباك المفاجئ.				
الدوخة ومشاكل الرؤية المفاجأة.				
صعوبة في الفهم فجأة.				
كثرة الرغبة في التبول، والشعور بالحرقة أثناء التبول، والبول الغائم.				
فجأة لم أستطع الوصول إلى حقيبتي لأنني لمأتمكن من تحريك ذراعي اليمنى. بدأت الترويل من زاوية فمي. عندما حاولت طلب المساعدة من زوجي، لم تكن الكلمات صحيحة.				
صداع مفاجئ حاد دون سبب معروف.				
مشكلة مفاجئة في التحدث.				
ألم في الصدر يدوم أكثر من بضع دقائق أو يزداد بشدة.				
لاحظت أنه ظل يغطي ويكشف عينيه ويومض. قال لي، "لا أستطيع أن أرى". بعد دقائق قليلة كان كل شيء على ما يرام مرة أخرى.				
ضعف مفاجئ للوجه خاصة على جانب واحد.				
ضعف مفاجئ في الذراع والوجه، خاصة من جانب واحد، مع صعوبة في التحدث.				
فجأة لم تتحرك ذراعي اليمنى.				
فقدان التنسيق المفاجئ				
